# A New Subclassification of pT4 Gastric Cancers According to the Width of Serosal Invasion

**DOI:** 10.1371/journal.pone.0068042

**Published:** 2013-06-27

**Authors:** Yue Kang, Feng Wang, Hongliang Zu, Zhenyu Yang, Yingwei Xue

**Affiliations:** 1 Department of Gastroenterologic Surgery, The Tumor Hospital of Harbin Medical University, Harbin, China; 2 Department of General Surgery, The Second Affiliated Hospital of Harbin Medical University, Harbin, China; 3 Department of General Surgery, The Affiliated Hospital of Inner Mongolia Medical University, Hohhot, China; University of Verona, Italy

## Abstract

**Objective:**

The purpose of this study was to propose a novel subclassification of pT4 gastric cancers according to the width of serosal changes and to investigate the validity and clinical utility of this subclassification as a predictor of prognosis.

**Methods:**

A total of 780 pT4 stage gastric cancer patients classified according to the 7th American Joint Committee on Cancer (AJCC) staging system were reviewed. Clinicopathologic features were compared between patients with narrow serosal changes (nSE), wide serosal changes (wSE) and invasions of adjacent structures (SI). Prognostic factors were evaluated by univariate and multivariate analyses. The 7th AJCC and novel pT4 subclassification were compared for prognostic performance using the linear trend chi-square test, likelihood ratio chi-square test, and Akaike information criterion (AIC) in the Cox regression analysis.

**Results:**

The appropriate serosa infiltrate cutoff value was 8 cm. Most of the evaluated clinicopathologic features significantly differed between nSE and SI cancers. Only 3 factors were significantly different between wSE and SI cancers. The 5-year survival rates for patients with the novel pT4a and pT4b cancers were 47.2% and 14.52%, respectively, while they were 41.66% and 16.34% for the 7th AJCC pT4a and pT4b cancers, respectively. The novel pT4 subclassification had better discriminatory ability, monotonicity of gradients, and homogeneity and had smaller AIC values compared with the 7th AJCC pT4.

**Conclusions:**

It is reasonable to subclassify pT4 to pT4a (nSE) and pT4b (wSE/SI) because the novel pT4 subclassification had more potential to identify the different prognoses for patients with gastric cancer.

## Introduction

Although the prognosis of patients with gastric cancer continues to improve, it remains the fourth most common malignant tumor and the second leading cause of cancer-related death worldwide [1]–[3]. It is widely accepted that the most important prognostic indicators in gastric cancer are the depth of wall invasion (pT) and the status of lymph node metastasis (pN) [4]–[6]. Therefore, the accurate categorization of invasive depth and lymph node metastasis or the optimization of the pT and pN categories is fundamentally critical for determining the disease extent, providing guidance for treatment planning, and predicting outcome [7].

The TNM classification of gastric cancer is one of the most commonly used staging systems. This system is accepted and maintained by the American Joint Committee on Cancer (AJCC). In 2010, the 7th AJCC TNM staging system was published [8]. Compared with the 6th, the 7th subdivided the pT1 category into pT1a (mucosal, M) and pT1b (submucosal, SM), reclassified the previous pT2a and pT2b categories as pT2 (muscularis propria, MP) and pT3 (subserosa, SS), and reclassified the former pT3 and pT4 categories as pT4a (perforates serosa, SE) and pT4b (invades adjacent structures, SI). Furthermore, the N classification was subdivided based on the number of metastatic lymph nodes. However, to the best of our knowledge, there is no consensus regarding the modification of the pT4 subclassification. Recently, some studies have shown significant differences between the survival curves of patients with stages T4a and T4b [9], [10]. By contrast, some other studies have reported no significant difference in prognosis between patients with stages pT4a and pT4b [11], [12]. For pT4a, the magnitude of serosal changes was highly related to the biological behavior of the tumor, and the prognosis for large areas of serosal changes was poor [13]–[15]. We hypothesized that tumors with large areas of serosal changes are similar to SI in biological behavior and prognosis and should be subclassified into the same subgroup of the pT4 stage to resolve the contradictions between previous studies.

In this study, we divided the width of serosal changes into wide serosal changes (wSE) and narrow serosal changes (nSE) and put forward a novel subclassification for pT4 as pT4a (nSE) and pT4b (wSE/SI) cancers. Furthermore, we investigated the validity and clinical utility of this subclassification as a predictor of prognosis.

## Patients and Methods

### Patients

From the archives of the Department of Gastroenterologic Surgery of the Tumor Hospital of Harbin Medical University, China, we identified all patients with histologically confirmed primary gastric adenocarcinoma who underwent curative gastrectomy between January 1997 and December 2007. A total of 969 patients with pT4 stage cancer (according to the 7th AJCC TNM staging system) were entered into a prospectively maintained database. All of the selected patients had undergone either total or partial gastrectomy plus a D2/D3 lymphadenectomy. Nodal dissection of the en bloc surgical specimen was performed by experienced surgeons in a standardized fashion (following the Japanese Research Society for Gastric Cancer guidelines) [16]. The surgical procedure was defined as a curative resection (R0, absence of residual tumor both macroscopically and microscopically). Postoperative mortality was defined as death within 30 days after surgery. The written informed consent had been obtained from all the patients, and this study was approved by the Research Ethics Committee of Harbin Medical University (Harbin, China). The study was retrospective.

The inclusion and exclusion criteria were defined as follows. Patients were included when histology confirmed an adenocarcinoma of the stomach and the date of death or survival data were available. Patients were excluded using the following criteria: histologically identified tumor of the esophagogastric junction; incomplete histopathologic data; synchronous malignancies or gastric stump cancer; fewer than 15 retrieved nodes; and definitive M1 classification based on the 7th AJCC TNM staging system. Among the potential participants, 57 underwent R1 or R2 resection, defined as microscopic or macroscopic remnant tumor, 26 died <30 days after resection, 35 had distant lymph node metastases (retropancreatic, mesenteric, duodenohepatic ligament or para-aortic lymph node), and 71 had unavailable clinical and histopathologic data. Thus, 189 patients were excluded. Of the remaining 780 patients, 518 (66.4%) were classified as nSE, 95 (12.2%) as wSE, and 167 (21.4%) as SI.

### Measurement of the Width of Serosal Invasion

The resected stomach was opened along the greater or lesser curvature to clearly expose the whole serous membrane. The maximum dimension of macroscopic serosal changes was defined as the width of serosal invasion for further analyses. In the gastric wall of the proximal one third that was not covered by serosa, the magnitude of perigastric fat infiltration was regarded as the width of serosal invasion. The width was presented as an average of values measured by 2 surgeons independently.

### Follow-up

The postoperative follow-up included clinical and laboratory examinations every 3 months for the first 2 years after surgery at our outpatient department and then every 6 months for 3 years and yearly thereafter. The check-up items included a physical examination, tumor marker studies, gastroendoscopy, chest radiography, and abdominal computed tomography or ultrasonography. The survival duration was calculated from the time of surgery to death or the last follow-up date (December 31, 2011). The median follow-up duration was 52.2 months (range 3.8 months to 119.4 months).

### Adjuvant Chemotherapy

Adjuvant chemotherapies were administered to patients with good performance status who gave their informed consent. A total of 273 patients were treated with postoperative chemotherapy. Three different chemotherapy regimens were used as follows: first, 5-fluorouracil (5-FU) 600 mg/m^2^ intravenous bolus injection on days 1, 8, 29 and 36,doxorubicin 30 mg/m^2^ on days 1 and 29, and mitomycin-C 10 mg/m^2^ on day 1,every 8 weeks (n = 46); second, oxaliplatin 85 mg/m^2^ as a 2-hour intravenous infusion on day 1, and leucovorin 200 mg/m^2^ as a 2-hour intravenous infusion followed by bolus 5-FU 400 mg/m^2^ and a 22-hour intravenous infusion of 5-FU 600 mg/m^2^ on days 1 and 2,every 2 weeks (n = 126); third, oxaliplatin 130 mg/m^2^ intravenous infusion over 2 h on day 1 plus oral capecitabine 1,000 mg/m^2^ twice daily on days 1–14, every 3 weeks (n = 101). In this study, no patients received neoadjuvant chemotherapy.

### Statistical Analysis

The chi-square test was used to compare the distributions of patient characteristics. The 5-year survival rates were estimated by the Kaplan-Meier method, and the differences among the levels of possible prognostic factors were compared by the log-rank test in a univariate analysis. Two separate multivariate analyses of prognostic factors related to overall survival were performed using the Cox proportional regression model.

The prognostic performance of the novel pT4 subclassification was compared with the 7th AJCC pT4 subclassification according to homogeneity, discriminatory ability, and monotonicity of gradients. The likelihood ratio chi-square test was used to measure homogeneity. The discriminatory ability and the monotonicity of gradients were assessed with the linear trend chi-square test. The Akaike information criterion (AIC) within the Cox proportional regression model was calculated to measure the discriminatory ability. A smaller AIC value represents better optimistic prognostic stratification [17]–[19]. All statistical tests were two tailed, and *P*-values less than 0.05 were considered to be statistically significant. The statistical analyses were performed using SAS software (version 9.1.3, SAS Institute, Cary, NC).

## Results

### Stratification of the Width of Serosal Changes

The mean ± SD of the serosal change width was 5.57±3.13 cm. Survival rates were calculated at each 1-cm interval to determine the appropriate serosal change width threshold. According to the Cox proportional hazard regression model, the highest chi-square score was considered the optimal cutoff point. Table 1 shows the stratification of the serosal change widths. The most significant difference in survival rates was at the threshold value of 8 cm (chi-square value = 68.498, hazard ratio = 2.814, *P*<0.0001). The patients were then divided into a narrow serosal changes group (nSE) and a wide serosal changes group (wSE).

**Table 1:** Chi-square score and hazard ratio according to the width of serosal changes calculated by Cox proportional hazard regression model.

Threshold	Chi-square	*P*	HR	95%CI
1	16.054	<.0001	4.617	2.185–9.758
2	34.614	<.0001	3.318	2.225–4.947
3	65.295	<.0001	3.079	2.344–4.045
4	64.571	<.0001	2.522	2.013–3.161
5	51.289	<.0001	2.130	1.732–2.619
6	47.696	<.0001	2.060	1.678–2.528
7	45.561	<.0001	2.128	1.709–2.649
8	**68.498**	<.0001	2.814	2.202–3.595
9	54.784	<.0001	2.947	2.214–3.924
10	51.097	<.0001	3.336	2.397–4.641
11	53.489	<.0001	4.506	3.010–6.746
12	39.326	<.0001	4.601	2.856–7.414
13	9.236	0.002	2.793	1.440–5.416
14	2.107	0.147	2.322	0.744–7.243

95% CI, 95% confidence interval.

HR, hazard ratio.

### Patient Characteristics

The clinicopathologic features of patients with nSE, wSE and SI cancers are shown in Table 2. As shown, there were significant differences in age, tumor size, resection type, longitudinal location, circumferential location, histologic type, lymphatic/venous invasion or lymph node metastasis between patients with nSE and SI cancers. The wSE and SI groups significantly differed in tumor size, longitudinal location and circumferential location.

**Table 2:** Clinicopathologic features of patients with nSE, wSE and SI gastric cancers.

	nSE(n = 518)		wSE(n = 95)		SI(n = 167)
Variable	n (%)	*P* *	n (%)	*P* *	n (%)
**Age (years)**		**0.0071**		0.1662	
<60	283(54.6)		55(57.9)		111(66.5)
≥60	235(45.4)		40(42.1)		56(33.5)
**Sex**		0.3786		0.327	
Male	376(72.6)		67(70.5)		127(76)
Female	142(27.4)		28(29.5)		40(24)
**Tumor size (cm)**		**<.0001**		**<.0001**	
<7	380(73.4)		6(6.3)		77(46.1)
≥7	138(26.6)		89(93.7)		90(53.9)
**Borrmann type**		0.7104		0.2544	
I/II	87(16.8)		10(10.5)		26(15.6)
III/IV	431(83.2)		85(89.5)		141(84.4)
**Resection type**		**<.0001**		0.067	
Subtotal	397(76.6)		40(42.1)		90(53.9)
Total	121(23.4)		55(57.9)		77(46.1)
**Longitudinal location**		**<.0001**		**0.0005**	
Lower	253(48.8)		11(11.6)		58(34.7)
Middle	60(11.6)		4(4.2)		9(5.4)
Upper	64(12.4)		7(7.4)		10(6)
Entire	141(27.2)		73(76.8)		90(53.9)
**Circumferential location**		**<.0001**		**<.0001**	
Other	486(93.8)		42(44.2)		134(80.2)
Encircling	32(6.2)		53(55.8)		33(19.8)
**Histologic type**		**0.0073**		0.982	
Differentiated	212(40.9)		28(29.5)		49(29.3)
Undifferentiated	306(59.1)		67(70.5)		118(70.7)
**Lymphatic/Venous invasion**		**<.0001**		0.145	
Negative	421(81.3)		35(36.8)		77(46.1)
Positive	97(18.7)		60(63.2)		90(53.9)
**Lymphnode metastasis**		**<.0001**		0.1293	
pN0	130(25.1)		3(3.1)		14(8.4)
pN1	95(18.3)		6(6.3)		19(11.3)
pN2	146(28.2)		20(21.1)		38(22.8)
pN3	147(28.4)		66(69.5)		96(57.5)

* Compared with SI cancers.

### Survival Analysis

**Figure 1:**
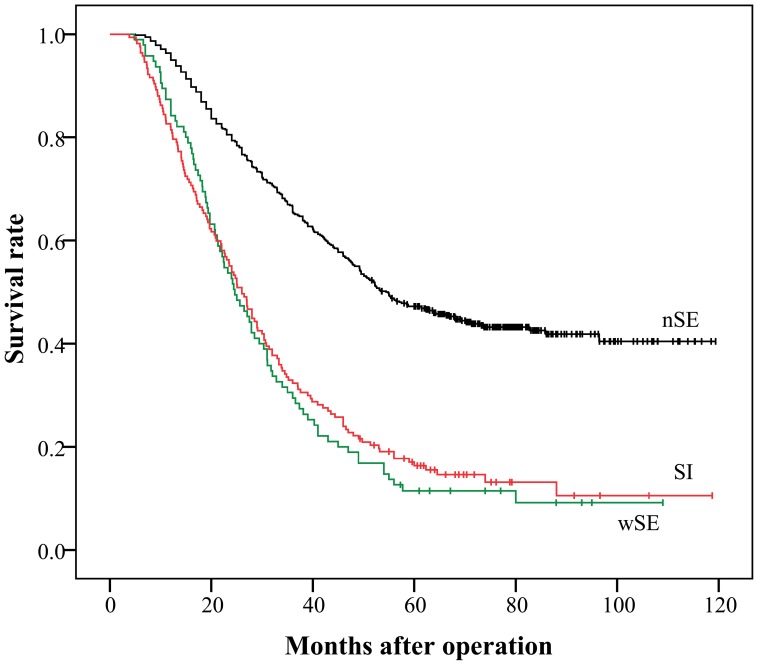
Kaplan-Meier survival curves of 780 patients with pT4 cancer stratified according to the width of serosal invasion. There was a significant difference in survival between nSE and SI cancers (*P*<0.0001), but not between wSE and SI cancers (*P* = 0.6084).

**Figure 2:**
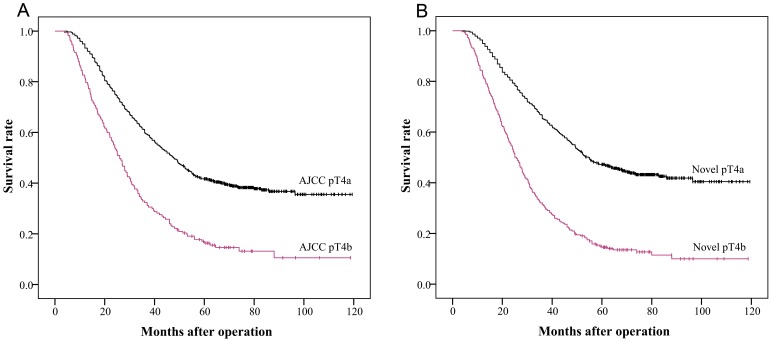
Kaplan-Meier survival curves using the AJCC and novel pT4 subclassification. (A) When the 7th AJCC pT4 subclassification applied, prognosis of pT4b stage cancers was significantly different with that of pT4a stage cancers (chi-square score = 64.487, P<0.0001). (B) When the novel pT4 subclassification applied, prognosis of pT4b stage cancers was significantly different with that of pT4a stage cancers (chi-square score = 125.694, P<0.0001).

Figure 1 shows the survival curves for the patients with nSE, wSE and SI cancers. There was a significant difference in survival between the nSE and SI groups (47.2% versus 16.34%, *P*<0.0001). However, the difference between the wSE and SI groups was not significant (11.37% versus 16.34%, *P* = 0.6084). The survival curves for the patients with the 7th AJCC classification and the novel pT4 subclassification are shown in Figure 2. The 5-year survival rates for patients with AJCC pT4a and pT4b stage cancers were 41.66% and 16.34%, respectively (*P*<0.0001) (Figure 2A), whereas the survival rates for the novel pT4a and pT4b stages were 47.2% and 14.52%, respectively (*P*<0.0001) (Figure 2B).

Univariate analysis showed that the significant prognostic factors associated with survival were tumor size, resection type, longitudinal location, circumferential location, histologic type, lymphatic/venous invasion, lymph node metastasis, adjuvant chemotherapy, AJCC pT4 stage, and the novel pT4 stage (Table 3). To evaluate the independent impacts of the 7th AJCC classification and the novel pT4 subclassification on overall survival, 2 separate multivariate Cox regression models were performed (one with the AJCC pT4 categories and one with the novel pT4 categories) (Table 4). The results demonstrated that the novel pT4 subclassification, lymph node metastasis and adjuvant chemotherapy remained independent prognostic factors.

**Table 3:** Univariate analysis of the prognostic factors for patients with pT4 gastric cancers.

Variable	n	5-year survival rate (%)	Chi-square	*P* value
**Age (years)**			0.745	0.388
<60	449	35.27		
≥60	331	37.62		
**Sex**			1.702	0.192
Male	570	34.95		
Female	210	39.84		
**Tumor size (cm)** *			43.480	**<.0001**
<7	457	45.61		
≥7	323	23.08		
**Borrmann type**			1.216	0.270
I/II	123	40.58		
III/IV	657	35.46		
**Resection type** *			18.921	**<.0001**
Subtotal	527	40.2		
Total	253	31.01		
**Longitudinal location** *			27.536	**<.0001**
Lower	322	43.94		
Middle	73	47.95		
Upper	81	34.33		
Entire	304	25.9		
**Circumferential location** *			23.321	**<.0001**
Other	662	38.83		
Encircling	118	21.83		
**Histologic type** *			5.943	**0.015**
Differentiated	289	40.68		
Undifferentiated	491	33.68		
**Lymphatic/Venous invasion** *			92.061	**<.0001**
Negative	533	44.71		
Positive	247	18.01		
**Lymph node metastasis** *			183.263	**<.0001**
pN0	147	74.05		
pN1	120	53.27		
pN2	204	28.64		
pN3	309	16.64		
**Chemotherapy** *			8.1010	0.0044
No	507	40.04		
Yes	273	51.28		
**Novel pT4 subclassification** *			125.694	**<.0001**
pT4a (nSE)	518	47.2		
pT4b (wSE/SI)	262	14.52		
**AJCC pT4 subclassification** *			64.487	**<.0001**
pT4a (SE)	613	41.66		
pT4b (SI)	167	16.34		

* Statistically significant (*P*<0.05).

**Table 4:** Multivariate analysis of the prognostic factors for patients with pT4 gastric cancers.

Variable	Chi-Square	*P* value	HR	95% CI
**Multivariate model with AJCC pT4 subclassification**				
Tumor size	3.8287	0.0504	1.301	1–1.693
Resection type	1.748	0.1861	1.169	0.927–1.474
Longitudinal location				
Lower				
Middle	1.1906	0.2752	0.811	0.557–1.181
Upper	0.0036	0.9522	0.989	0.688–1.422
Entire	0.5089	0.4756	0.902	0.679–1.198
Circumferential location	0.3364	0.5619	1.082	0.829–1.413
Histologic type	3.4563	0.063	1.195	0.99–1.442
Lymphatic/Venous invasion	1.644	0.1998	1.15	0.929–1.424
**Lymph node metastasis** *				
pN0				
pN1	9.8314	0.0017	1.852	1.26–2.722
pN2	53.3023	<.0001	3.536	2.519–4.964
pN3	91.1172	<.0001	5.018	3.603–6.988
**Chemotherapy** *	16.461	<.0001	0.675	0.558–0.816
**AJCC pT4 subclassification** *	44.7389	<.0001	1.994	1.629–2.441
**Multivariate model with Novel pT4 subclassification**				
Tumor size	0.2576	0.6118	1.07	0.823–1.391
Resection type	1.3143	0.2516	1.141	0.911–1.428
Longitudinal location				
Lower				
Middle	1.0346	0.3091	0.825	0.57–1.195
Upper	0.0274	0.8686	0.971	0.682–1.381
Entire	0.1503	0.6983	0.947	0.718–1.249
Circumferential location	0.6853	0.4078	0.893	0.683–1.168
Histologic type	3.6738	0.0553	1.2	0.996–1.447
Lymphatic/Venous invasion	3.5119	0.0609	1.22	0.991–1.501
**Lymph node metastasis** *				
pN0				
pN1	10.7941	0.001	1.906	1.297–2.801
pN2	51.3088	<.0001	3.459	2.463–4.858
pN3	80.7698	<.0001	4.602	3.299–6.419
**Chemotherapy** *	17.2113	<.0001	0.67	0.554–0.809
**Novel pT4 subclassification** *	51.4444	<.0001	2.066	1.694–2.519

* Statistically significant (*P*<0.05).

### Prognostic Performance

**Figure 3:**
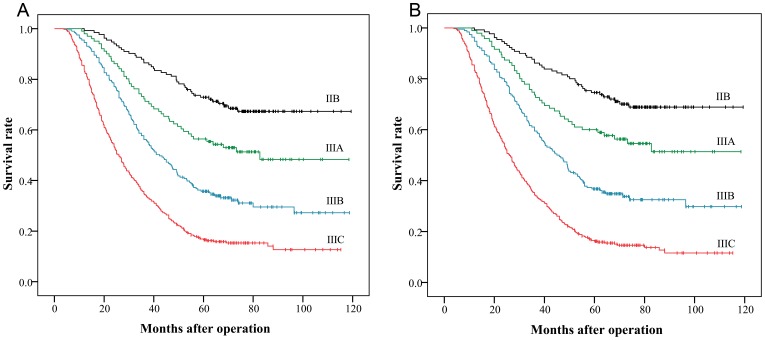
Kaplan-Meier survival curves using the 7th AJCC and novel TNM stage. (A) Prognostic performances of the 7th AJCC stage (linear trend chi-square score = 166.1493, likelihood ratio chi-square score = 182.3456, AIC = 6252.888). (B) Prognostic performances of the novel stage (linear trend chi-square score = 177.3985, likelihood ratio chi-square score = 198.6962, AIC = 6236.538).

The prognostic abilities of the 7th AJCC subclassification and the novel pT4 subclassification were assessed by the linear trend chi-square score, the likelihood ratio chi-square score, and the AIC tests are presented in Table 5. Compared with the 7th AJCC pT4 subclassification, the novel pT4 subclassification had better homogeneity (higher likelihood ratio chi-square score, 108.6238 versus 53.9337), discriminatory ability and gradient monotonicity (higher linear trend chi-square score, 103.6116 versus 53.3501). We further evaluated the performance of the novel and 7th AJCC pT4 subclassifications in the TNM staging system (Figure 3A, 3B). The results showed that the novel categories had better performance in the likelihood ratio chi-square score, the linear trend chi-square score and the AIC value than the 7th AJCC categories (Table 5).

**Table 5:** Comparison of the prognostic performances of the 7th AJCC and novel subclassifications.

Variable	Staging system	Linear trendchi-square*	Likelihood ratiochi-square†	AIC¶	Figure
**Stage**	AJCC stage	166.1493	182.3456	6252.888	**3A**
	Novel stage	177.3985	198.6962	6236.538	**3B**
**pT4 subclassification**	AJCC pT4	53.3501	53.9337	6381.300	**2A**
	Novel pT4	103.6116	108.6238	6326.610	**2B**

AIC, Akaike information criterion.

* Higher linear trend chi-square scores show better discriminatory ability and gradient monotonicity.

† A higher likelihood ratio chi-square score means better homogeneity.

¶ Smaller AIC values indicate better optimistic prognostic stratification.

## Discussion

The evaluation of cancer extent or stage at time of diagnosis is a key factor for defining a specific treatment and assessing the actual chances of cure. Accurate staging is necessary to describe the severity of an individual’s tumor, and it may provide clinicians with the means to determine prognosis and to compare groups of patients in new clinical trials. The AJCC TNM staging system is the most important tool for specific treatment and for assessing patient prognosis [20]. Specifically, the recently modified 7th TNM staging system hallmarks substantial changes for gastric cancer [21], [22]. Several studies have confirmed the prognostic accuracy of the new TNM staging system [7], [12], [23], but the validity of the division of pT4 lesions into pT4a (perforates serosa, SE) and pT4b (invades adjacent structures, SI) is unclear. Wang et al. [7] analyzed 1,503 gastric cancer patients who underwent surgical resection and concluded that the survival curves in each pT subgroup in the 7th edition staging system had excellent discriminatory abilities. By contrast, Hong et al. [12] analyzed 1,799 cases of gastric cancer from a Korean study and concluded that the survival curves for T4a and T4b cancers were not significantly different. We speculated that a new method for subclassifying pT4 cancers would be required to resolve the disagreement between previous studies.

It has been shown that the width of serosal invasion is closely correlated to the biological behavior of the gastric cancer, and is an independent prognostic factor in patients with pT4 tumors [13], [14]. Therefore, it might be a candidate for use in the TNM staging system. To date, there has been no formal proposal focused on the role of serosal invasion width in the gastric cancer staging system. Hence, in the current study, we proposed a novel pT4 subclassification according to the width of serosal invasion. Further, we investigated the effect of this subclassification on disease staging and patient prognosis.

Previous studies have demonstrated that, following the penetration of the serosal surface by cancer cells, the likelihood of the exfoliation of cancer cells from the lesions into the peritoneal cavity correlates with the magnitude of serosal invasion. Lesions with wide serosal infiltration indicate advanced local spread physically and exhibit increased aggressive biological behavior, resulting in cancer cell exfoliation from the serosal surface, these behaviors are significantly different from those of narrow serosal infiltration [13], [14]. In theory, all gastric cancers invading adjacent structures (pT4b) have micrometastases in the peritoneal cavity. Therefore, the 7th AJCC pT4 category may not make a rigid distinction between SE gastric cancer and SI gastric cancer. In the current study, the critical serosa cutoff point for narrow/wide serosal changes was 8 cm. The width of serosal invasion was divided into nSE (serosal changes ≤8 cm) and wSE (serosal changes >8 cm). nSE cancers significantly differed with SI cancers for the majority of the evaluated clinicopathologic features, while only 3 clinicopathologic features differed between wSE and SI cancers. These observations support the hypothesis that the biological behavior and prognosis of wSE cancers is more homogeneous with SI cancers and should be subclassified into the same pT category subgroup.

Several studies have reported that the prognosis of patients with wide serosal changes is significantly poorer than those with narrow serosal changes [24], [25]. In accordance with these studies, we found that the 5-year survival rates for patients with nSE was significantly higher than those with wSE cancers. However, the difference of the 5-year survival rates between the wSE and SI groups was not significant. These results suggest that it is reasonable to subclassify pT4 cancers as pT4a (nSE) and pT4b (wSE/SI). In addition, our present results confirmed that postoperative adjuvant chemotherapy significantly improved overall survival compared with surgery alone. The adjuvant chemotherapy was an independent prognostic factor in patients with pT4 tumors by further multivariate analysis. Since the distribution of patients received adjuvant chemotherapy in the new pT4a stage was similar to that in the pT4b stage, there was no bias by an unequal distribution of adjuvantly treated patients between the two stages.

When the overall survival rates were compared between the pT4a and pT4b stages, the 7th AJCC pT4 categories and the novel pT4 categories consistently significantly differed, indicating that the 2 categories were valuable for prognostic assessment. However, in a univariate analysis, the log-rank chi-square score associated with the novel pT4 categories was larger than that of the 7th AJCC pT4 categories. These results further indicate that the novel pT4 categories could provide a more detailed subclassification and a more homogenous prognosis than the 7th AJCC pT4 categories. In this study, the novel and 7th AJCC pT4 subclassifications were highly correlated. To decrease the impact of bias on the survival rates, 2 separate multivariate models were performed to avoid multicollinearity. The results demonstrated that the novel pT4 subclassification could discriminate 2 subsets of patients with greater differences in prognosis than the 7th AJCC pT4 subclassification, indicating that the novel pT4 subclassification was the most important independent factor for gastric cancer prognosis.

Ueno and colleagues [17] have analyzed the prognostic performance of the staging systems by homogeneity, discriminatory ability and monotonicity of gradients and demonstrated the following: 1) the difference in survival time is small among patients within the same stage (homogeneity); 2) compared with this difference, patients in different stages have much greater survival time differences (discriminatory ability); 3) the mean survival time for patients with earlier stages of cancer is longer than patients in later stages (monotonicity of gradients). In the current study, the novel pT4 subclassification demonstrated better homogeneity, discriminatory ability and monotonicity of gradients than the 7th AJCC pT4 subclassification. We further validated the performance using the TNM staging system. The results revealed that the novel stages had better performance than the 7th AJCC stages for the likelihood ratio chi-square score and the linear trend chi-square score. Furthermore, it had a smaller AIC value, indicating a less information loss when predicting outcome, representing the optimistic prognostic stratification.

Our study has some limitations. First, the determination of the width of serosal changes is mainly dependent on subjective measuring by surgeons, which may introduce bias due to the multiple surgeons included in the study. Second, the study was performed at a single center in China, and the sample size was relatively small compared to the worldwide gastric cancer collaboration database. To solve this problem, a multi-center, large-scale study concerning this new subclassification of pT4 gastric cancers should be conducted to further confirm our results.

In conclusion, the present study demonstrates that wSE cancers had similar clinicopathologic characteristics and prognosis to SI cancers. Therefore, it is reasonable to subclassify pT4 cancers as pT4a (nSE) and pT4b (wSE/SI). The novel pT4 subclassification had a greater potential to identify the different prognoses. We propose that the current category should be modified to better represent the prognosis for patients with pT4 gastric cancers.
